# Cohort Profile: The Tokyo Adolescent Mental Health Study (TAMHS)

**DOI:** 10.2188/jea.JE20250338

**Published:** 2026-10-05

**Authors:** Rui Zhou, Satoshi Yamaguchi, Atsushi Nishida, Satoru Tsuihiji, Fumiharu Togo, Tsukasa Sasaki

**Affiliations:** 1 Department of Physical and Health Education, Graduate School of Education, The University of Tokyo, Tokyo, Japan; 2 Unit for Mental Health Promotion, Research Center for Social Science & Medicine, Tokyo Metropolitan Institute of Medical Science, Tokyo, Japan; 3 Secondary School attached to the Faculty of Education, The University of Tokyo, Tokyo, Japan

**Keywords:** mental health, adolescent, twins, cohort, longitudinal study

## Abstract

The Tokyo Adolescent Mental Health Study (TAMHS) is an ongoing, school-based longitudinal cohort designed to elucidate the developmental trajectories of mental health among adolescents in Japan and to inform evidence-based strategies for promoting adolescent well-being. Since its inception in 2009, TAMHS has annually tracked adolescents aged 12–18 from a Tokyo secondary school known for its tradition of enrolling twins and triplets. Participants are followed for up to 6 years. As of 2024, 2,435 adolescents (1,211 boys, 1,196 girls, 28 unspecified), including 551 twins and triplets (22%), have been enrolled. This unique sample composition allows for rigorous comparisons between singletons and multiples to examine the impact of birth plurality on neurodevelopmental and mental health trajectories. High annual retention (96.2–99.2%) ensures the integrity of longitudinal analyses. Data are collected through self-report questionnaires covering demographics, physical and mental health status, daily habits and lifestyle behaviors, social relationships, behavioral problems, healthcare access, and social and environmental determinants of health. The study has yielded important insights into associations between adolescent lifestyle behaviors and mental health outcomes.

## WHY WAS THE COHORT SET UP?

Adolescence is a critical period for mental health, yet the prevalence of mental disorders is increasing worldwide.^1^^,^^2^ Approximately 15% of adolescents are estimated to experience a mental disorder, but most do not receive adequate care.^3^ In Japan, despite a declining birthrate, the number of patients under 20 years with mental disorders reached 599,000 in 2020, representing more than a fivefold increase since 1999.^4^ Understanding the determinants of adolescent mental health, including biological, environmental, and perinatal influences, is essential for informing early intervention and prevention strategies. While numerous epidemiological cohorts have investigated adolescent mental health, most have been restricted to singleton births. This limitation hampers our understanding of how early-life birth characteristics, such as preterm birth and low birth weight, may shape mental health trajectories, especially in biologically vulnerable populations like twins and triplets.^5^

Twin and triplets, who are at higher risk of being born preterm and with low birth weight, may face additional neurodevelopmental and psychiatric challenges.^6^ Emerging evidence suggests that they may have higher rates of attention-deficit/hyperactivity disorder, autism spectrum disorder, and mood disorders compared to singletons.^7^^–^^9^ However, studies on adolescent mental health have rarely examined these risks within a broader developmental context. Existing twin studies primarily focus on genetic heritability, often overlooking the potential biological, developmental, and environmental vulnerabilities associated with being a twin or triplets itself, particularly in relation to mental health.

The Tokyo Adolescent Mental Health Study (TAMHS) was established to comprehensively examine adolescent mental health in a longitudinal, school-based cohort that includes both singleton and multiple-birth individuals. Following adolescents aged 12–18 years annually for up to 6 years, the study tracks changes in mental and physical health along with other developmental factors. While TAMHS primarily focuses on adolescent mental health more broadly, twins and triplets make up over 22% of the study, providing a unique opportunity to investigate whether multiple-birth individuals face distinct challenges compared to singletons. This sample composition allows for an additional exploration of whether being a twin or triplet constitutes an independent risk factor for psychiatric and neurodevelopmental outcomes. Moreover, conducted in Japan—a context of high academic pressure and distinct social norms—TAMHS offers valuable insights into adolescent mental health beyond Western-centric research. By capturing diverse developmental patterns in this unique sociocultural setting, it also contributes to a more nuanced global understanding of adolescent trajectories, including those of multiple-birth individuals, an often understudied population.

## WHO IS IN THE COHORT

TAMHS cohort study follows adolescents from a secondary high school in Tokyo, Japan. The study school is a national school with decades of history and locates in the central urban area of Tokyo. It consists of junior and senior high students from grade 7 (ages 12–13 years) to grade 12 (ages 17–18 years), annually enrolls approximately 120 seventh grade students (60 boys and 60 girls) through an entrance examination, and therefore around 720 students registered in the school each year. In addition, the school's annual admissions include a specific number of twins and triplets for research purposes.

The study protocol was reviewed and approved by the Ethics Committee of the Life Science Committee of the University of Tokyo (*#15–128*) and by the Research Department of the school. Each year, written information outlining the study’s aims, methods, and voluntary nature was provided to guardians of all students at the same time, who could opt their children out by returning a form or contacting the school. Data collection was conducted in the school using self-report questionnaires. On the day of the survey, students were informed of their right to decline participation, either by leaving the classroom or submitting a blank questionnaire. To ensure privacy, each questionnaire was distributed with an envelope, allowing participants to seal their responses upon completion and prevent others from viewing their answers. The surveys were completed anonymously, with participants only providing their school ID numbers, enabling longitudinal follow-up.

Between 2009 and 2024, a total of 2,435 adolescents (1,211 boys, 1,196 girls, and 28 of unspecified sex), including 551 twins or triplets, participated in the study, with consistently high annual participation rates (96.2–99.2%; Table 1). The small proportion of non-participants primarily consisted of students who were absent on the survey day due to illness or other personal reasons (eg, studying abroad or taking a leave of absence).

**Table 1.** Annual survey participation numbers and rates of TAMHS

Survey years	2009	2010	2011	2012	2013	2014	2015	2016	2017	2018	2019	2020	2021	2022	2023	2024
Number of participants	350	698	705	699	707	707	706	706	701	701	701	703	708	697	688	682
Total number of students	354	707	712	715	714	717	713	713	709	710	712	709	714	715	715	705
Participation rates (%)	98.9	98.7	99.0	97.7	99.0	98.6	99.0	99.0	98.9	98.7	98.5	99.2	99.2	97.5	96.2	96.7

TAMHS, Tokyo Adolescent Mental Health Study.

## HOW OFTEN HAVE THEY BEEN FOLLOWED UP?

The survey has been conducted annually since 2009, typically in June (with the 2020 survey delayed to August due to coronavirus disease 2019–related restrictions). The first wave (wave 1) included 354 participants from three grades (7th, 9th, and 11th). From the second wave onwards, all current students have been invited to participate annually. Each year, a new cohort of 7th-grade students is recruited, and participants are followed up annually until the end of 12th grade, resulting in a 6-year follow-up period for each cohort (see Figure 1).

**Figure 1.**
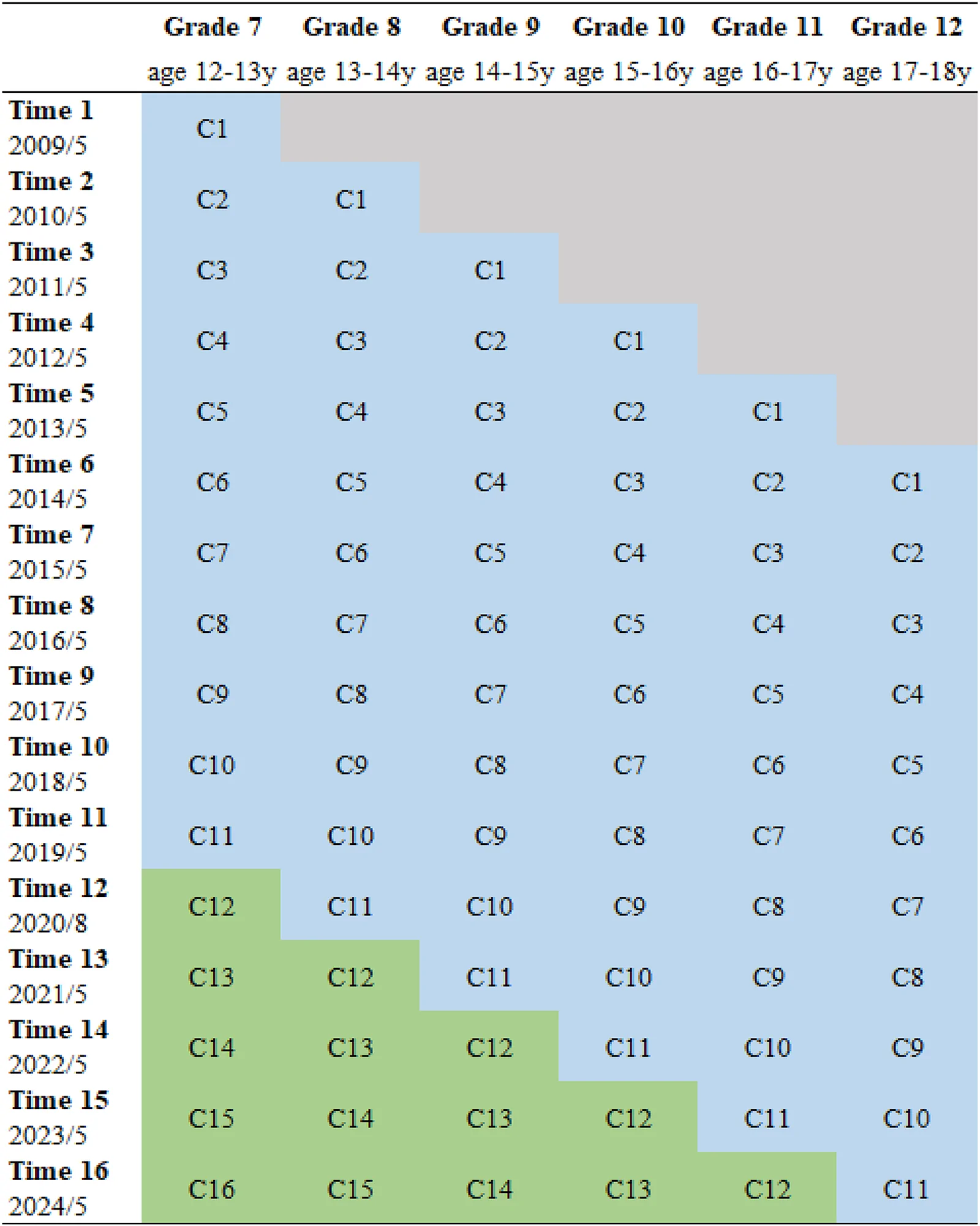
Cohort structure in the TAMHS study design. C, cohort; TAMHS, Tokyo Adolescent Mental Health Study. Color coding reflects follow-up status: blue indicates participants who completed the full 6-year follow-up, green indicates those currently under follow-up, and gray indicates those who completed follow-up of less than 6 years.

As of 2024, 11 cohorts (2009–2019) have completed follow-up, while 5 cohorts (2020–2024) are ongoing. The most recent cohort (Cohort 16) was established in 2024, and data collection for Cohort 17 (2025) is currently ongoing.

## WHAT HAS BEEN MEASURED?

Table 2 summarizes all measures and the waves in which they were assessed. Broadly, the survey covers six domains: (i) demographic information (eg, age, sex, twin or triplet status, household composition); (ii) mental health and psychosocial factors (eg, depressive/anxiety symptoms, suicidal ideation, self-harm, psychotic-like experiences); (iii) physical health and development (eg, pubertal stage, body mass index, somatic symptoms, participation in physical activities); (iv) daily lifestyle behaviors (eg, sleep, diet, smoking/alcohol/substance use, travel time to school); (v) social relationships and behavioral risks (eg, number of confidantes, help-seeking sources, bullying involvement, violent behavior); and (vi) digital media use and regulation (eg, smartphone use after lights-off, late-night media use, family rules on digital device use). Missing data were generally low for all measures, with rates ranging from 0% to 5% for key mental health variables (eTable 1).

**Table 2.** Overview of TAMHS data collection

	T1	T2	T3	T4	T5	T6	T7	T8	T9	T10	T11	T12	T13	T14	T15	T16
***Demographic information***																
Age	P	P	P	P	P	P	P	P	P	P	P	P	P	P	P	P
Sex	P	P	P	P	P	P	P	P	P	P	P	P	P	P	P	P
Birthday	P	P	P	P	P	P	P	P	P	P	P	P	P	P	P	P
Sibling situation	P	P	P	P	P	P	P	P	P	P	P	P	P	P	P	P
Twin or triplet	P	P	P	P	P	P	P	P	P	P	P	P	P	P	P	P
Household composition	P	P	P	P	P	P	P	P	P	P	P	P	P	P	P	P
***Mental health and psychosocial factors***																
Depression/anxiety symptoms	P	P	P	P	P	P	P	P	P	P	P	P	P	P	P	P
Suicidal ideation	P	P	P	P	P	P	P	P	P	P	P	P	P	P	P	P
Self-harm	P	P	P	P	P	P	P	P	P	P	P	P	P	P	P	P
Psychotic-like experiences	P	P	P	P	P	P	P	P	P	P	P	P	P	P	P	P
Stress/mental health concerns	P	P	P	P	P	P	P	P	P	P	P	P	P	P	P	P
Mental well-being						P	P	P	P	P	P	P	P	P	P	P
Personality traits						P	P	P	P	P	P	P	P	P	P	P
Willingness to seek professional help	P	P	P	P	P	P	P	P	P	P	P	P	P	P	P	P
***Physical health and development***																
Pubertal stage				P	P	P	P	P	P	P	P	P	P	P	P	P
Body mass index	P	P	P	P	P	P	P	P	P	P	P	P	P	P	P	P
Somatic symptoms	P	P	P	P	P	P	P	P	P	P	P	P	P	P	P	P
Participation in physical activities										P	P	P	P	P	P	P
School club participation			P	P	P	P	P	P	P	P	P	P	P	P	P	P
Perceived athletic competence			P	P	P	P	P	P	P	P	P	P	P	P	P	P
***Daily lifestyle behaviors***																
Sleep duration	P	P	P	P	P	P	P	P	P	P	P	P	P	P	P	P
Sleep habits						P	P	P	P	P	P	P	P	P	P	P
Sleep quality			P	P	P	P	P	P	P	P	P	P	P	P	P	P
Smoking, alcohol, and substance use	P	P	P	P	P	P	P	P	P	P	P	P	P	P	P	P
Breakfast frequency			P	P	P	P	P	P	P	P	P	P	P	P	P	P
Diet experience	P	P	P	P	P	P	P	P	P	P	P	P	P	P	P	P
Travel time to school									P	P	P	P	P	P	P	P
Study time outside school						P	P	P	P	P	P	P	P	P	P	P
Daily bathing/shower duration						P	P	P	P	P	P	P	P	P	P	P
Daily grooming/beauty duration						P	P	P	P	P	P	P	P	P	P	P
Household chores						P	P	P	P	P	P	P	P	P	P	P
After-school stop frequency						P	P	P	P	P	P	P	P	P	P	P
***Social relationships and behavioral risks***																
Number of confidantes	P	P	P	P	P	P	P	P	P	P	P	P	P	P	P	P
Help-seeking sources	P	P	P	P	P	P	P	P	P	P	P	P	P	P	P	P
Bullying involvement	P	P	P	P	P	P	P	P	P	P	P	P	P	P	P	P
Violent behavior	P	P	P	P	P	P	P	P	P	P	P	P	P	P	P	P
Experience of domestic violence	P	P	P	P	P	P	P	P	P	P	P	P	P	P	P	P
***Digital media use and regulation***																
Mobile phone use	P	P	P	P	P	P	P	P	P	P	P	P	P	P	P	P
Smartphone use after lights-off	P	P	P	P	P	P	P	P	P	P	P	P	P	P	P	P
Late-night media use		P	P	P	P	P	P	P	P	P	P	P	P	P	P	P
Family rules on digital device use									P	P	P	P	P	P	P	P

TAMHS, Tokyo Adolescent Mental Health Study.

Note: T1 to T16 correspond to the years 2009 to 2024.

Mental health variables were assessed using a combination of validated questionnaires and single-item measures. Depression/anxiety symptoms were assessed using the Japanese version of General Health Questionnaire–12 (GHQ-12), with higher scores indicating greater psychological distress over the past month.^10^ Psychotic-like experiences (PLEs) occurring in the past 6 months were assessed using five items from the schizophrenia section of the Japanese version of the Diagnostic Interview Schedule for Children (DISC-C).^11^ Mental well-being in the past 2 weeks was assessed using the World Health Organization-5 Well-Being Index, with higher scores indicating better well-being.^12^ Personality traits were assessed using the Big Five Inventory-10.^13^ Other mental health-related items were assessed using single self-report questions, as commonly used in large-scale epidemiological surveys.

Participant characteristics across survey waves are summarized in eTable 2. The demographic composition of the cohort remained stable over time, with a balanced sex distribution and consistent patterns across key socioeconomic and psychosocial variables that have been reported in our previous publications.^14^^–^^20^

## WHAT HAS IT FOUND?

To date, published findings from the TAMHS cohort (waves 1–11, completed cohorts) have provided important insights into adolescent development, particularly in relation to sleep behaviors, PLEs, and mental health outcomes.

### Sleep disturbances and mental health

TAMHS data consistently show that sleep disturbances, including short sleep duration (<7 hours/night) and delayed bedtimes, are associated to mental health issues, such as depression, anxiety, and PLEs. These associations are robust when examined using quantitative thresholds and standard statistical approaches. For example, longer sleep duration (8–10 hours/night) may mitigate the adverse effects of low physical activity (<1 hour/day of moderate-to-vigorous activity) on mental health, whereas sufficient physical activity can partially offset the risks of inadequate sleep.^14^ Each 1-hour delay in bedtime predicts a 0.22-point increase in GHQ-12 scores 1 year later, indicating worsening psychological distress.^18^ Habitual short sleep duration is also a significant predictor of PLEs: compared with 8–9 hours/night, the risk of PLEs increases 1.8-fold with 5–6 hours of sleep and nearly 6-fold with <5 hours of sleep.^17^ These findings were derived from multiple regression analyses in cross-sectional data,^14^ multivariate latent growth and autoregressive cross-lagged models in longitudinal data,^18^ and cross-lagged panel modeling capturing bidirectional associations,^17^ with adjustment for relevant covariates.

### PLEs and mental health

Longitudinal analyses of TAMHS indicate that worsening depressive and anxiety symptoms are prospectively associated with increased risk of PLEs, suggesting that common mental health problems may contribute to subclinical psychotic symptoms in adolescents.^19^ PLEs have also been demonstrated to be bidirectionally associated with severe mental health outcomes, including suicidal ideation and self-harm.^16^ In addition to the association with internalizing problems, PLEs have been linked to externalizing behaviors. For instance, our recent study has demonstrated a bidirectional longitudinal relationship between PLEs and violent behavior (especially violence towards objects), particularly in girls, indicating potential gender-specific pathways.^15^

### Twin studies and psychosocial factors

Findings from TAMHS also provide insights into the role of genetic and environmental factors in adolescent mental health. A study comparing twins and singletons found that twins exhibited lower levels of PLEs and psychological distress, suggesting potential protective factors in twin dynamics.^20^ The same study further indicated that psychological distress was linked to irregular sleep patterns, bullying experiences, and low perceived social support, emphasizing the importance of stable sleep routines and supportive social environments.^20^

### Overview of key outcomes

In addition to these specific associations, previously published findings synthesize evidence on key outcomes. Psychological distress, assessed by the GHQ-12 (0–12), showed mean scores ranging from approximately 1.7 to 4.0 between Grades 7 and 12, depending on the analytic sample and study. In parallel, suicidal ideation was reported by about 4–10% of students, while the prevalence of PLEs was estimated at approximately 6.5–10.8% during the same period.^16^^–^^19^ Other outcomes, including sleep duration, self-harm, and violent behavior, have also been reported in TAMHS publications, highlighting the breadth of psychosocial and behavioral domains covered in this cohort.

Taken together, these findings illustrate the multifaceted interplay among sleep, PLEs, psychosocial factors, and mental health outcomes, and underscore the value of longitudinal cohort studies in identifying modifiable risk factors and informing early preventive interventions.

### Future research prospects

Future research of the TAMHS cohort will aim to clarify the developmental pathways linking early psychosocial and behavioral experiences to later mental health outcomes. These efforts will include mapping longitudinal trajectories of depression/anxiety symptoms, suicidal ideation, self-harm, PLEs, and other key mental health outcomes into early adulthood; examining lifestyle and social-contextual factors—such as bullying, family and peer support, twins and triplets status, physical activity, and sleep habits—as potential mediators and moderators of these trajectories; and applying advanced longitudinal methods (eg, growth curve modeling, random intercept cross-lagged models, latent pathway analyses) to strengthen causal inference and capture bidirectional dynamics of adolescent mental health.

## WHAT ARE THE MAIN STRENGTHS AND WEAKNESSES?

This prospective multicohort study advances longitudinal research on adolescent health through several principal strengths. First, the rigorous methodological design combines annual structured data collection across physical, psychological, behavioral, and environmental domains with follow-up rates consistently exceeding 96%, effectively minimizing attrition bias and enhancing statistical power to identify developmental trajectories. Second, the multilevel analytical approach allows simultaneous examination of individual-level changes from early to late adolescence and period effects, the latter through comparisons of successive cohorts exposed to evolving environmental factors (eg, the proliferation of digital media). Third, the frequent assessments of TAMHS across key developmental stages over an extended period allow for a nuanced analysis of the dynamic interplay between lifestyle factors and health outcomes, offering valuable guidance for age-specific interventions. Finally, the inclusion of a substantial number of twin and triplet participants enables robust within-family comparisons, disentangling genetic and environmental influences on adolescent development. Longitudinal follow-up further enhances this design, allowing for the examination of how these influences interact over time to shape adolescent mental health and behavior.

This study has several limitations. First, the reliance on self-reported measures for mental health and behavioral outcomes may introduce recall bias and social desirability bias. Second, the generalizability of findings may be limited, as participants were recruited from a single school with potentially low socioeconomic diversity, which may not fully represent rural populations or minority groups. Lastly, minor revisions to measurement tools (eg, the social media use scale) may limit the longitudinal comparability of certain constructs, even though core measurement properties remain consistent.

## CAN I GET HOLD OF THE DATA? WHERE CAN I FIND OUT MORE?

The TAMHS data are managed and maintained by the Health Education Laboratory, Graduate school of Education, The University of Tokyo. Researchers interested in collaboration are welcome to send specific research proposals to the research committee. The initial contact point for collaboration is [psytokyo577@gmail.com].

## SUPPLEMENTARY MATERIAL

eTable 1. Missing data rates (%) for mental health items in the TAMHS cohort

eTable 2. Participant characteristics of the TAMHS cohort (2009–2024)

## References

[1] Solmi M, Radua J, Olivola M, . Age at onset of mental disorders worldwide: large-scale meta-analysis of 192 epidemiological studies. Mol Psychiatry. 2022 Jan 2;27(1):281–295. 10.1038/s41380-021-01161-7 34079068 PMC8960395

[2] Copeland WE, Wolke D, Shanahan L, Costello EJ. Adult functional outcomes of common childhood psychiatric problems. JAMA Psychiatry. 2015 Sep 1;72(9):892–899. 10.1001/jamapsychiatry.2015.0730 26176785 PMC4706225

[3] World Health Organization. Adolescent mental health. WHO Fact sheet. 2021. Available from: https://www.who.int/news-room/fact-sheets/detail/adolescent-mental-health. Accessed 23.09.2025.

[4] Ministry of Health, Labour and Welfare. Summary of the 2020 Patient Survey. 2021. Available from: https://www.mhlw.go.jp/toukei/list/10-20.html. Accessed 23.09.2025.

[5] Wainstock T, Yoles I, Sergienko R, Sheiner E. Twins vs singletons—long-term health outcomes. Acta Obstet Gynecol Scand. 2023 Aug 25;102(8):1000–1006. 10.1111/aogs.14579 37186304 PMC10377983

[6] Hiersch L, Okby R, Freeman H, . Differences in fetal growth patterns between twins and singletons. J Matern Fetal Neonatal Med. 2020 Aug 2;33(15):2546–2555. 10.1080/14767058.2018.1555705 30501543

[7] Anderson PJ, de Miranda DM, Albuquerque MR, . Psychiatric disorders in individuals born very preterm / very low-birth weight: an individual participant data (IPD) meta-analysis. EClinicalMedicine. 2021 Dec;42:101216. 10.1016/j.eclinm.2021.101216 34901794 PMC8639417

[8] Ho A, Todd RD, Constantino JN. Brief Report: Autistic traits in twins vs. non-twins? A preliminary study. J Autism Dev Disord. 2005 Feb;35(1):129–133. 10.1007/s10803-004-1040-8 15796128

[9] Dooley N, Healy C, Cotter D, Clarke M, Cannon M. The persistent effects of foetal growth on child and adolescent mental health: longitudinal evidence from a large population-based cohort. Eur Child Adolesc Psychiatry. 2023 Oct 21;32(10):2067–2076. 10.1007/s00787-022-02045-z 35861893 PMC10533650

[10] Goldberg DP, Rickels K, Downing R, Hesbacher P. A comparison of two psychiatric screening tests. Br J Psychiatry. 1976;129(7):61–67. 10.1192/bjp.129.1.61 938806

[11] Costello EJ, Edelbrock CS, Costello AJ. Validity of the NIMH diagnostic interview schedule for children: a comparison between psychiatric and pediatric referrals. J Abnorm Child Psychol. 1985;13:579–595. 10.1007/BF00923143 4078188

[12] World Health Organization. Wellbeing measures in primary health care/the DepCare Project: report on a WHO meeting: Stockholm, Sweden, 12–13 February 1998. Available from: https://iris.who.int/handle/10665/349766. Accessed 16.12.2025.

[13] Rammstedt B, John OP. Measuring personality in one minute or less: a 10-item short version of the Big Five Inventory in English and German. J Res Pers. 2007 Feb;41(1):203–212. 10.1016/j.jrp.2006.02.001

[14] Ogawa S, Kitagawa Y, Fukushima M, . Interactive effect of sleep duration and physical activity on anxiety/depression in adolescents. Psychiatry Res. 2019 Mar;273:456–460. 10.1016/j.psychres.2018.12.085 30684792

[15] Zhou R, Foo JC, Nishida A, Ogawa S, Togo F, Sasaki T. The association between psychotic-like experiences and violent behavior in adolescents: a cross-lagged longitudinal study. Eur Child Adolesc Psychiatry. 2025 Jun 11;34(12):3769–3777. 10.1007/s00787-025-02770-1 40498326 PMC12743062

[16] Zhou R, Foo JC, Nishida A, Ogawa S, Togo F, Sasaki T. Longitudinal relationships of psychotic-like experiences with suicidal ideation and self-harm in adolescents. Eur Child Adolesc Psychiatry. 2024 Jun 23;33(6):1977–1985. 10.1007/s00787-023-02299-1 37740799 PMC11211151

[17] Zhou R, Foo JC, Yamaguchi S, . The longitudinal relationship between sleep length and psychotic-like experiences in adolescents. Psychiatry Res. 2022 Nov;317:114893. 10.1016/j.psychres.2022.114893 36244161

[18] Tochigi M, Usami S, Matamura M, . Annual longitudinal survey at up to five time points reveals reciprocal effects of bedtime delay and depression/anxiety in adolescents. Sleep Med. 2016;17:81–86. 10.1016/j.sleep.2015.08.024 26847979

[19] Yamasaki S, Usami S, Sasaki R, . The association between changes in depression/anxiety and trajectories of psychotic-like experiences over a year in adolescence. Schizophr Res. 2018;195:149–153. 10.1016/j.schres.2017.10.019 29055569

[20] Oshima N, Nishida A, Fukushima M, . Psychotic-like experiences (PLEs) and mental health status in twin and singleton Japanese high school students. Early Interv Psychiatry. 2010;4(3):206–213. 10.1111/j.1751-7893.2010.00186.x 20712725

