# Supplementary material for: Cohort Profile: The Tokyo Adolescent Mental Health Study (TAMHS)
Source: J Epidemiol. 2026 Oct 5;36(10):328–33. doi: 10.2188/jea.JE20250338 (PMC13562593; doi:10.2188/jea.JE20250338)
Supplement: Supplementary file 1 [file je-36-328-s001.pdf]

eTable 1. Missing data rates (%) for mental health items in the TAMHS cohort

Variables/Year	2009	2010	2011	2012	2013	2014	2015	2016	2017	2018	2019	2020	2021	2022	2023	2024
Depression/anxiety symptoms	0.3	0.3	2.0	1.0	0.1	2.8	2.1	1.8	0.7	2.0	0.7	0.6	0.1	1.0	0.3	0.0
Suicidal ideation	0.0	0.1	1.6	0.3	0.0	3.2	2.4	2.7	1.1	1.1	0.6	1.3	2.7	5.0	3.3	4.5
Self-harm	1.1	2.1	8.7	1.1	2.3	2.9	2.3	2.8	1.1	1.4	1.0	1.6	0.4	1.1	0.9	1.0
Psychotic-like experiences	0.3	0.1	0.4	0.4	0.0	1.8	1.4	1.6	0.4	1.1	0.9	1.1	0.3	1.3	0.1	0.4
Violence to objects	1.1	0.6	0.4	0.7	0.3	1.5	1.3	2.0	0.4	0.9	0.1	0.6	3.5	0.7	0.7	0.3
Interpersonal violence	1.1	0.3	0.3	1.2	0.9	2.0	2.1	1.7	0.4	0.4	0.4	0.6	0.3	0.7	1.0	0.4

TAMHS, Tokyo Adolescent Mental Health Study.

eTable 2. Participant characteristics of the TAMHS cohort (2009–2024)

Characteristic/Year	2009	2010	2011	2012	2013	2014	2015	2016	2017	2018	2019	2020	2021	2022	2023	2024
Female, n (%)	171 (48.9)	344 (49.3)	352 (50.0)	349 (50.0)	356 (50.5)	354 (49.5)	349 (49.2)	354 (50.0)	350 (50.0)	350 (50.0)	346 (49.6)	344 (49.4)	341 (48.9)	337 (49.4)	338 (49.2)	339 (50.2)
Twins/triplets, n (%)	81 (23.1)	159 (22.8)	146 (20.7)	149 (21.3)	146 (20.7)	145 (20.3)	136 (19.2)	142 (20.1)	144 (20.7)	135 (19.3)	129 (18.5)	134 (20.7)	125 (18.7)	118 (17.8)	119 (18.2)	119 (18.2)
Weekday sleep duration, hours, mean (SD)	6.9 (1.1)	6.8 (1.2)	6.9 (1.1)	6.9 (1.1)	6.8 (1.0)	6.9 (1.1)	6.9 (1.1)	6.9 (1.1)	6.9 (1.0)	6.9 (1.1)	7.0 (1.1)	6.7 (1.2)	6.8 (1.2)	6.8 (1.2)	6.9 (1.2)	7.0 (1.2)
Weekend sleep duration, hours, mean (SD)	-	-	8.6 (1.3)	8.5 (1.3)	8.4 (1.4)	8.4 (1.3)	8.4 (1.3)	8.4 (1.3)	8.4 (1.4)	8.4 (1.5)	8.4 (1.4)	8.1 (1.5)	8.3 (1.6)	8.3 (1.6)	8.5 (1.6)	8.6 (1.7)
Depression/anxiety symptoms, mean (SD)	3.1 (3.1)	3.4 (3.0)	2.9 (3.0)	2.6 (2.8)	2.9 (3.0)	2.5 (2.7)	2.7 (2.9)	2.5 (2.7)	2.6 (2.9)	2.4 (2.8)	2.4 (2.8)	2.9 (3.1)	2.6 (2.9)	2.5 (2.7)	2.8 (2.9)	2.6 (2.7)
Suicidal ideation, n (%)	44 (12.6)	-	61 (8.7)	43 (6.2)	47 (6.6)	41 (5.7)	44 (6.2)	48 (6.8)	56 (8.0)	51 (7.3)	56 (8.0)	67 (9.5)	66 (9.4)	56 (7.8)	53 (8.1)	56 (7.3)
Self-harm, n (%)	21 (6.1)	49 (7.0)	45 (6.4)	50 (7.2)	49 (6.9)	48 (6.7)	35 (4.9)	41 (5.8)	37 (5.3)	46 (6.6)	40 (5.7)	39 (5.6)	49 (7.1)	80 (12.1)	51 (7.7)	61 (9.4)
Psychotic-like experiences, n (%)	78 (22.3)	135 (19.3)	59 (8.4)	62 (8.9)	53 (7.5)	58 (8.1)	50 (7.0)	45 (6.3)	50 (7.1)	53 (7.6)	72 (10.3)	64 (9.2)	58 (8.2)	72 (10.5)	75 (10.9)	74 (10.9)

SD, standard deviation; TAMHS, Tokyo Adolescent Mental Health Study.
